# Improving the Conversion Ratio of QDSCs via the Passivation Effects of NiS

**DOI:** 10.3390/nano14110905

**Published:** 2024-05-22

**Authors:** Edson Leroy Meyer, Mojeed Adedoyin Agoro

**Affiliations:** 1 Fort Hare Institute of Technology, University of Fort Hare, Private Bag X1314, Alice 5700, Eastern Cape, South Africa; emeyer@ufh.ac.za; 2 Department of Chemistry, University of Fort Hare, Private Bag X1314, Alice 5700, Eastern Cape, South Africa

**Keywords:** passivation, heterostructure, semiconductors, stability, QDSSCs

## Abstract

To revolutionize the photochemical efficiency of quantum dots sensitized solar cells (QDSSCs) devices, herein, a passivation of the cells with multilayer material has been developed for heterojunctions TiO_2_/NiS/MnS/HI-30/Pt devices. In this study, NiS and MnS were deposited on a photoanode for the first time as passivated photon absorbers at room temperature. The adoption of NiS as a passisvative layer could tailor the active surface area and improve the photochemical properties of the newly modified cells. The vibrational shifts obtained from the Raman spectra imply that the energy change is influenced by the surface effect, giving rise to better electronic conductivity. The electrochemical stability and durability test for the N/M-3 device slows down and remains at 8.88% of its initial current after 3500 s, as compared to the N/M-1 device at 7.20%. The disparity in charge recombination implies that both the outer and inner parts of the nanoporous material are involved in the photogeneration reaction. The hybridized N/M-3 cell device reveals the highest current density with a low potential onset, indicating that power conversion occurs more easily because photons tend to be adsorbed easily on the surface of the MnS. The Nyquist plot for N/M-1 and N/M-3 promotes the faster transport of electrolytic ions across the TiO_2_/NiS/MnS, providing a good interaction for the electrolyte. The I-J Value of 9.94% shows that the passivation with the NiS layer promotes electron transport and enhances the performance of the modified cells. The passivation of the TiO_2_ layer with NiS attains a better power conversion efficiency among the scant studies so far on the surface passivation of QDSCs.

## 1. Introduction

International interest in the development of high-performance and cost-friendly solar cells with clean energy generation and sustainable realization is of great use in resolving the energy catastrophe [1,2]. Progress has been made in the most recent three decades with various solar cells [3,4,5,6,7], with the most recent one being perovskite solar devices. With a certified power conversion above 22% [8] for perovskite solar cells with a close gap to commercial silicon monocrystalline solar cells, perovskite’s major shortfall in achieving practical application and commercialization is its instability barrier compared to that of quantum dot-sensitized solar cells (QDSSCs), which have better stability. The attention QDSSCs have been given in recent years is not only due to their good stability alone but also due to their numerous advantages, like their easy fabrication, multiple exciton generation, and the tunable band of their semiconductor quantum dots (QDs) [9,10,11]. Despite the promising possessing merits of these semiconductor devices, their theoretical values of QDSSCs power conversion efficiency (PCE) are still not attained. To solve these issues, various pathways have been adopted to improve the PCE of QDSSCs in two steps. First, through interfacial alteration to lower trap-assisted charge recombination, and second, by controlling the semiconductor band gap to a QD size in order to absorb the entire solar spectrum. QDs such as CdS [12], Ag_2_S [13], CdSe [14], PbSe [15], PbS [16], and SnS [10,17] have been used as photon absorbers in QDSSCs.

**Figure 1:**
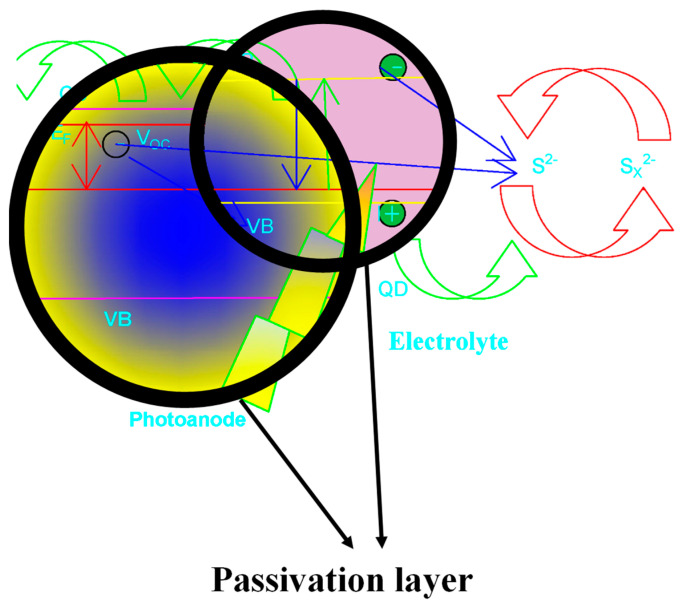
Passivation of the NiS-MnS layer in QDSC.

Among these sensitizers, SnS is one of the most promising QD materials; it has been explored in QDSSC applications with a prospective PCE. An ideal sandwich QDSSCs buildup involves a photoanode like TiO_2_ as an electron transport material, polysulfide electrolyte, QD sensitizer, and counter electrode [18], with several hetero-interfaces in solar cell devices, as highlighted above. Therefore, more attention should be given to the interface during the engineering build-up of QDSSCs to reduce charge recombination and further improve the PCE of the solar cells. The passivation of the photonaode interface or surface electrode devices is an effective pathway for treating or modifying the cell device [9]. The effectiveness of the passivation of the surface of QD absorbers in eliminating or reducing surface traps has been used to enhance the separation of photogenerated charge carriers. Thus, the interface of the passivative material with the wideband-gap photoanode nanostructure materials should be considered. Ideally, QD photosensitizers are coated on the surface of semiconductors like TiO_2_ through different techniques, with few chances to control the interface between them. In order to reduce carrier recombination, the passivation of the photoanode nanostructure with a wide band gap with other materials with a smaller band gap is a promising strategy. Two types of passivation layers have been reported through thin insulating passivation layers of metal oxides, such as ZrO_2_ [19], Al_2_O_3_ [20], HfO_2_ [21], and SiO_2_ [22,23]. The second one is through the sulfide semiconductor passivation layer, which increases the QD loading mass, reduces surface defects, and traps charge recombination. The recombination of charge carriers at the photoanode/electrolyte interfacing posed a significant threat to the QDSSC performance. A straightforward arrangement might be fixing up any pathway for the charge to recombine at the interface. Charge recombination is facilitated by QDs’ limited surface coverage of the large band gap metal oxide layer. As a result, a passivation layer is deposited to protect the photoanode surface. The layers helped to reduce charge recombination at the QD/electrolyte and TiO_2_/QD interfaces, as seen in Figure 1 [24].

Myoung et al. [25] obtained an improved efficiency of 4.32% by passivating a layer of QDSCs using CuS. Antonio et al. [26], on the other hand, increased the conversion efficiency to 2.21% by directly growing PbS on the surface of TiO_2_ nanostructured electrodes. Luo et al. used MnS/ZnO photoanodes to enhance the efficiency to 3.70% for QDSSCs [9]. Nevertheless, the passivation approach to semiconductor wide-band-gap materials research is still in its very early phase, which implies that more studies are necessary [27]. The introduction of metal sulfide from a single-source molecular precursor as an efficient co-catalyst on TiO_2_ before sensitization with MnS as an absorber could enhance the electrochemical activity. Nickel sulfide (NiS) is a significant candidate with excellent reducing activity for Sn^2−^ reduction, earth abundance, and a low cost [28]. At present, the preparation of NiS as a passivative material and MnS as a photon absorber by the two-step process could lead to a high conversion output [29,30,31,32]. Also, the injection of NiS may effectively trap charge recombination but may also promote the migration of holes between the electrolyte and the QDs [9]. In this work, a NiS passivation layer is introduced between mesoporous TiO_2_ before loading MnS QD sensitizers, rather than being coated on the surface of QD sensitizers, as is generally done. To fabricate the NiS-MnS passivative layer using the Equations (1) and (2), MnS was dissolved in distilled water and reacted at room temperature. The K_SP_ of MnS is 1.4 × 10^−15^, which is substantially higher than that of NiS, which is 3 × 10^−21^, showing that MnS is not produced by ion exchange between Mn^2+^ and NiS but rather through the combination of Mn^2+^ and S^2−^. Due to the dynamic equilibrium of the dissolution, NiS is generated in the solution through an ion exchange between the dissolved Ni^2+^ and MnS, resulting in the NiS-MnS layer [31,32,33].

(1) $${Mn}^{2+}+S^{2-}\;⟶MnS\;$$

(2) $$MnS+{Ni}^{2+}⟶NiS+{Mn}^{2+}$$

## 2. Materials and Methods

### 2.1. Materials and Methods

Water, NiS, and MnS are from aromatic *N*,*N*-dibenzyl-*N*-*p*-anisidineldithiocarbamato Ni(II) complexes and *N*-anil-*N*-*p*-anisdtc Mn(II) complexes. Complete testing kits containing platinum fluorine-doped tin oxide (FTO), TiO_2_ FTO, HI-30 polysulfide electrolyte, gaskets, masks, and hot seals were purchased from Solaronix.

### 2.2. Synthesis of the NiS QDs and MnS

**Figure 2:**
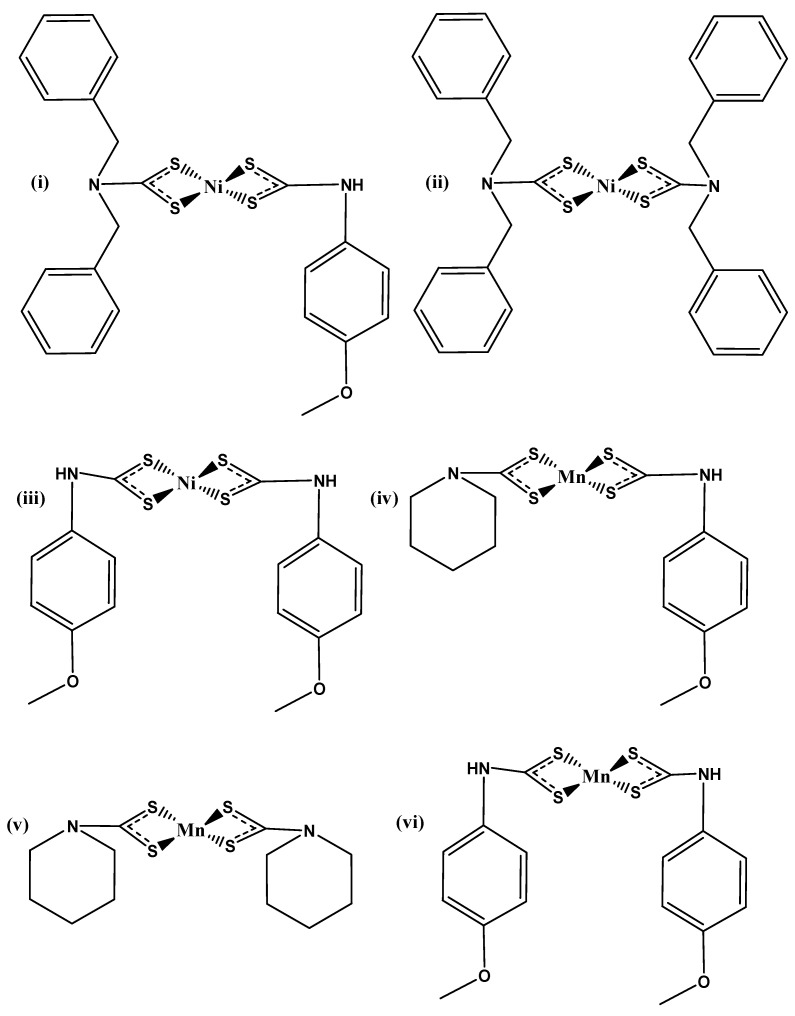
Dithiocarbamate complexes (**i**–**vi**) used for the synthesis of NiS and MnS as N/M-1, N/M-2, and N/M-3 in the present study.

*bis(N,N-dibenzyl-N-p-anisidineldithiocarbamato)* nickel(II) as Ni[*N*,*N*-benz-*N*-*p*-anisidineldtc] was used to synthesize NiS as NiSa, while Ni[*N*,*N*-benzldtc] and Ni[*N*-*p*-anisldtc] were used under similar conditions to fabricate NiSb and NiSc. This was used as a passivative layer for the assembled thin film. MnS QDs were used as the photosensitizers synthesized from Mn[*N*-anil-*N*-*p*-anisdtc], Mn[*N*-piperdtc], and Mn[*N*-*p*-anisdtc] as MnS_1, MnS_2, and MnS_3, as seen in Figure 2. Both metal sulfides were previously reported [31,32]. This was used in the current study as NiSa/MnS_1 as N-M-1, NiSb/MnS_2 as N-M-2, and NiSc/MnS_3 as N-M-3.

### 2.3. Assembly of DSSCS

The synthesis of NiS and MnS used for this study has been reported [31,32]. The TiO_2_ films were first immersed in 0.1 g of NiS mixed with distilled water for 10 h and then rinsed with distilled water and dried in the air. Subsequently, the films were immersed in a 0.1 g MnS solution in distilled water for 10 h to allow for the loading of both materials on the TiO_2_ [7]. The corresponding films were denoted as TiO_2_/NiS/MnS as (N-M-1), TiO_2_/NiS/MnS as (N-M-2), and TiO_2_/NiS/MnS as (N-M-3). The QD-sensitized TiO_2_ electrode and the Pt CE were assembled and sealed in layers using a transparent 50 mm hot melt sealing sheet. An HI-30 electrolyte is injected through a pinhole made in the CE. The cells were left for a few hours to complete the diffusion of the electrolyte within the photo-anode, and the cells were tested under one-sun illumination (AM 1.5 G, 100 mW cm^−2^).

### 2.4. Physical Characterizations

X-ray diffraction (XRD) phase identification was carried out using Bruker D8 Advance with monochromatic CuKα (λ = 1.5406 Å) radiation in the 2θ scale range of 20°−80°, while the high-resolution transmission electron microscopy (HRTEM) size diameter, selected area electron diffraction (SAED), and lattice fringes of the as-prepared cell devices were taken from JEOL HRTEM-2100. A ZEISS EVOLS 15 scanning electron microscope joined with energy dispersive X-ray spectroscopy were used to obtain the images and spectra of the scanned electron microscopy (SEM) and electron dispersive X-ray (EDX). The absorption spectra of the modified DSSC devices were observed using a Perkin Elmer Lambda 25 UV-Vis spectrometer. A Keithley 2400 Model 4066884, C32 instrument with an intensity of light of 1000 Wm^−2^ was employed to measure the current vs. voltage of the cells. A Gamry 10101E/ZRA Reference 3000 electrochemical workstation with a standard three-electrode setup consisting of working electrodes, a saturated calomel reference electrode, and a Pt counter electrode was used to study the interfacial charge transport of the prepared DSSCs devices through an EIS-Nyquist plot. Linear scanning voltammetry (LSV), cyclic voltammetry (CV), and chronoamperometry (CA) were evaluated on the three samples at a scan rate of 10 mV s^−1^ to assess the effect of the heterostructure size and morphology on the loading process of the cells. The Fourier transform infrared spectrum of the modified cells was evaluated using FTIR-Perkin Elmer Spectrum1 for functional group identification.

## 3. Results

### 3.1. XRD, Raman, and FTIR Analysis

**Figure 3:**
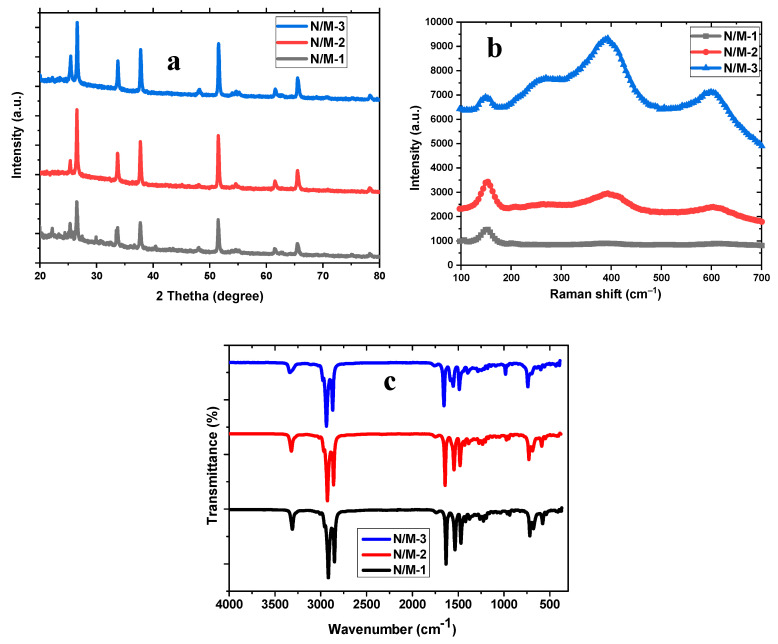
X-ray diffraction patterns (**a**), Raman spectra (**b**), and FTIR spectra (**c**) of N/M-1, N/M-2, and N/M-3 cells.

X-ray diffraction analysis was employed for the characterization of N-M-1, N-M-2, and N-M-3 modified electrodes to identify their crystallinity and phase composition, as seen in Figure 3a. The patterns show quite similar diffraction patterns for the three samples prepared from different molecular precursors. The XRD peaks are indexed to (100), (002), and (012) for MnS at 25.75, 26.51, and 37.72 and (002) and (201) for NiS at 33.70 and 65.44. This matches well with the hexagonal phase of MnS indexed according to PDF 96-153-8837 and the hexagonal NiS phase (PDF 96-153-8625). The broadening peaks reveal the crystallite sizes at 18.02 nm, 40.51 nm, 32.42 nm, 41.30 nm, 41.30 nm, and 41.31 nm for N-M-1, N-M-2, and N-M-3. The Scherrer formula was used to calculate the peaks at (002) 26.51 and (002) 33.70 for hexagonal MnS and NiS, as shown below.

(3) $$D=\frac{k\lambda }{\beta cos\theta }$$

where D is the crystal size, *k* is a constant, typically 0.9, λ = 0.15406 nm is the wavelength of the X-ray, *β* is the FWHM, and θ is the diffraction angle. The formation of metastable sulfur-rich phases is attainable with the use of tertiary and secondary amines, and the results of this study are consistent with previous literature on single-source molecular precursors [34,35,36]. The Raman spectral profiles of N-M-1, N-M-2, and N-M-3 modified electrodes are shown in Figure 3b, which reveals a Raman vibrational band at 608 cm^−1^, indexed to the Ti–O bond vibration. Raman shifts at 397 cm^−1^ are linked to the Mn–S vibration bond of the MnS photon absorber. Additional Raman peaks at 159 and 264 cm^−1^ match well with the Ni–S vibration bond of NiS. These vibrational shifts connote that the energy change is influenced by the surface effect, which gives rise to better electronic conductivity. The Raman and FTIR results authenticate the heterostructure phase of the three modified devices [37,38]. Figure 3c displays the modified N/M-1, N/M-2, and N/M-3 cell device FTIR spectra for a single phase. The band stretching modes for the C-H are detected at 2919 and 2929 cm^−1^. The peak linked to the N-H stretching is observed at 3400 cm^−1^ of the free amines. The peak for C-N was found within 1633–1472 cm^−1^, while the peak for C-S peaks at 720 cm^−1^ [39]. The vibration peaks at 581 and 413 cm^−1^ correlate to the presence of M-S metal with sulfur atom coordination, which is similar to the literature [31,32].

### 3.2. HRTEM and SAED Analysis

**Figure 4:**
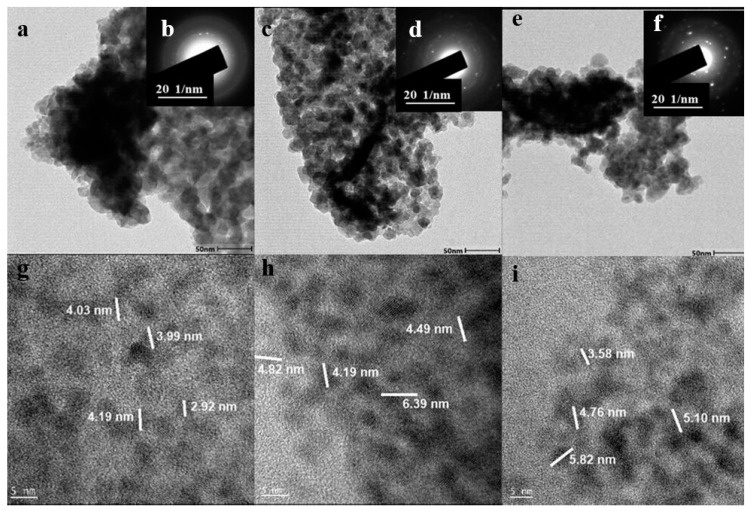
TEM (**a**,**c**,**e**), SAED (**b**,**d**,**f**), and HRTEM (**g**–**i**) of N/M-1, N/M-2, and N/M-3 cells.

The HRTEM images presented in Figure 4g–i endorse the SEM results showing a porous spherical device with a high surface area, which can be linked to the sulfur presence supported by the EDS 

The QDs size distributions within the range of 2.92–6.39 nm show the polycrystalline nature represented by the SAED pattern of the three samples; the diffraction spots correspond to (002) MnS and NiS. These are in good agreement with the XRD results in Figure 3a, which further correlated the structures of these samples with a synergetic effect, resulting in the high conversion efficiency of the as-prepared devices [40].

### 3.3. SEM/EDS Analysis

**Figure 5:**
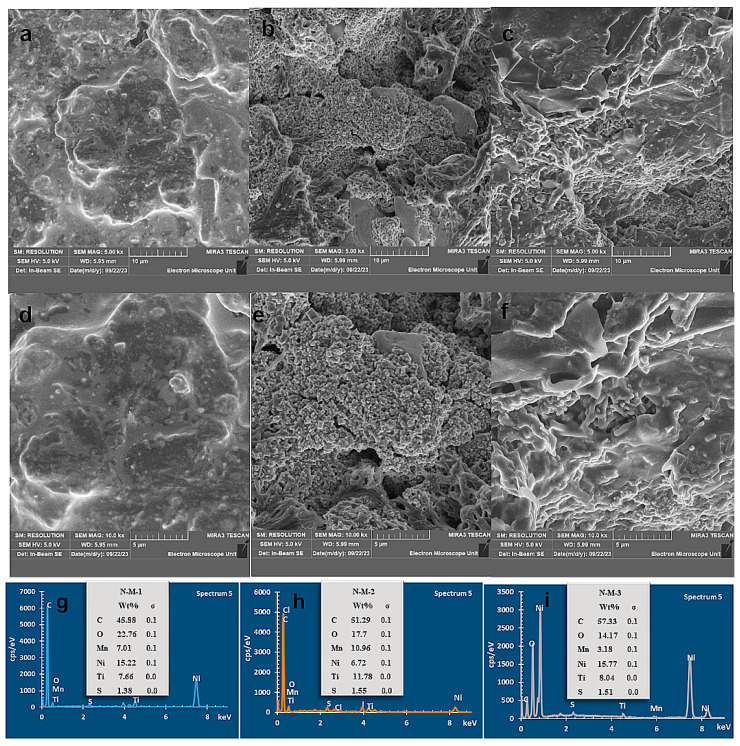
SEM (**a**–**f**) and EDS (**g**–**i**) of N/M-1, N/M-2, and N/M-3 cells.

To understand the molecular precursor effect on the morphology of TiO_2_/NiS/MnS electrode nanostructures, we investigated this effect at a fixed reaction temperature and reaction time, as seen in Figure 5a–f. The SEM image of the N/M-1 electrode shows an irregular spherical morphology, which is beneficial for contact between the electrolyte and active electrode material surface during the electrochemical process [41]. SEM images of the particles prepared from N/M-3 at fixed temperatures show the synthesis of coalesced spherical nanoparticles [42]. The N/M-2 consists of nanoparticles, but in non-homogenous, irregular shapes [43]. EDS confirms the elemental composition of Ni, Mn, S, and C in the entire cells, and the TiO_2_ layer was observed (Figure 5g–i) [38].

### 3.4. UV-Vis and Taucs Plot Analysis

**Figure 6:**
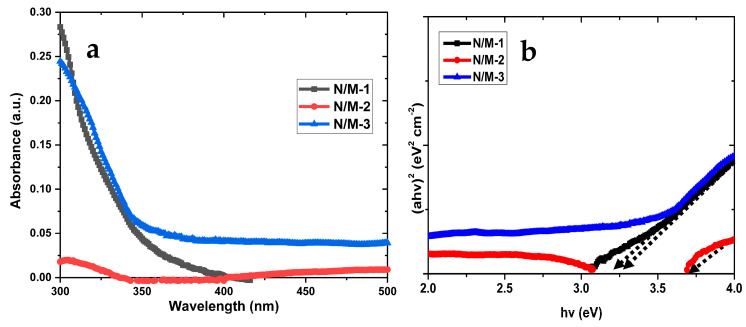
UV-Vis (**a**) and Taucs plot (**b**) of N/M-1, N/M-2, and N/M-3 cells. The black dotted arrow is the extrapolation line.

The effects of passivation on the optical characteristics of the modified electrodes with multilayers are studied through the UV-Vis spectrometer technique. The absorption spectra of N/M-1, N/M-2, and N/M-3 cell devices are illustrated in Figure 6a. The N/M-2 device gives an absorption peak at a wavelength less than 350 nm due to the charge transfer from the valence to the conduction band. The N/M-1 and N/M-3 cells’ increase in absorption could be due to an increase in the thickness of the electrodes [44,45]. The increase in absorption can also be linked to the electronic transition within the defect levels of TiO_2_ and the band structure [45]. Moreover, extensions in the absorption edge imply a good interaction between the metal ion grain and TiO_2_ [29]. The *E_g_* of TiO_2_ depends strongly on the grain size and the defects in the TiO_2_ band gap [44]. The *E_g_* of N/M-1, N/M-2, and N/M-3 cell devices (see Figure 6b) is calculated using the formula below.

(4) $$Eg=\frac{1240}{\lambda }eV$$

The *E_g_* of N/M-1, N/M-2, and N/M-3 is 3.25, 3.6, and 3.18 eV, respectively. The disparity in the *E_g_* values could be due to the carbon chain length of the molecular precursors used in the formation of metal sulfides. This change influences the energy levels of the cells by shifting the Fermi energy, leading to the narrowing of the band gap energy [44,45].

### 3.5. EIS and Nyquist Plots Analysis

**Figure 7:**
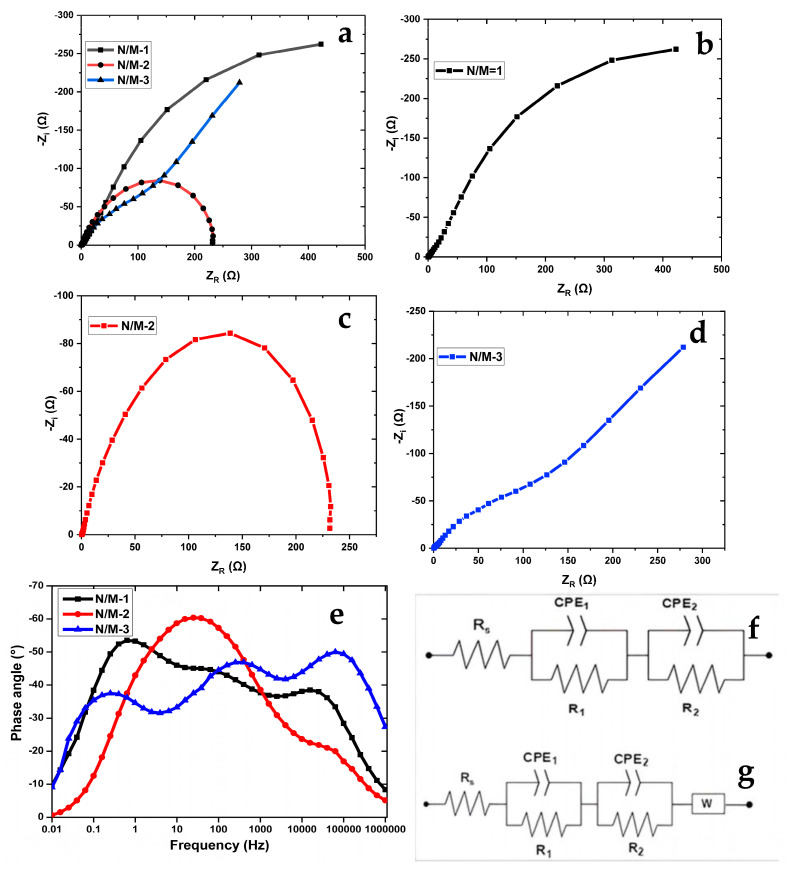
EIS Nyquist (**a**–**d**), Bode plot (**e**), and Equivalent model circuit of N/M-1, N/M-2 (**f**), and N/M-3 cells (**g**).

Nyquist plots from EIS tests for the three solar cell devices are shown in Figure 7a–d.

The Nyquist plots shown in Figure 7a–d show that the reactive portion of the devices N/M-1 and N/M-3 is significantly larger than the plot for N/M-2. This indicates that N/M-1 and N/M-3 both have a slower or more resistive charge transfer process compared to N/M-2. This can be confirmed by the Bode plot in Figure 7e, where it can be observed that the capacitive component across the main device body peaks relatively around the same values, particularly across the low-frequency region 1Hz<f<1kHz). This excludes the Warburg effect f<1kHz seen on N/M-1 and N/M-3. Since the impedance circuit of this region is equivalent to $R_{2}∥\;Z_{CPE2}$ and the capacitive effect is relatively the same, it shows that the higher impedance values observed from the Nyquist plot for N.M-1 and N/M-3 are mainly due to the resistive component of the charge transfer process [46].

The Bode plot shown in Figure 7e for the N/M-2 hybridized electrode shows the lowest resistance for electron transfer. The lower impedance values measured at the high-frequency region of the Nyquist plot for the devices N/M-1 and N/M-3 indicate the faster transport of electrolytic ions across the homogeneous TiO_2_/NiS/MnS electrode, which provides a good working surface for the iodolite HI-30 electrolyte and facilitates their interaction. [47,48]. The model adopted for the extraction of impedance and the capacitive value are obtained using Equations (5) and (6), which are similar to those in the literature [49,50,51].

(5) $$Z_{T}=R_{s}+R_{1}∥\;Z_{CPE1}+R_{2}∥Z_{CPE2}+Z_{W}$$

where $R_{s},\;R_{1},\;R_{2},\;Z_{CPE1},\;Z_{CPE2},\;and\;Z_{W}$ are the series resistance of the whole device measured directly from the Nyquist plot, estimated resistances, constant phase element impedance, and Warburg impedance for time-constant regions 1 and 2, as indicated by the Bode plot, respectively.

The Warburg element’s impedance can further be calculated using

(6) $$Z_{W}=\frac{\sigma }{\sqrt{2}\pi ƒ}\;–j\frac{\sigma }{\sqrt{2}\pi ƒ}$$

where σ is the Warburg coefficient and f is the frequency of the Warburg region.

### 3.6. LSV and CV Curve Analysis

**Figure 8:**
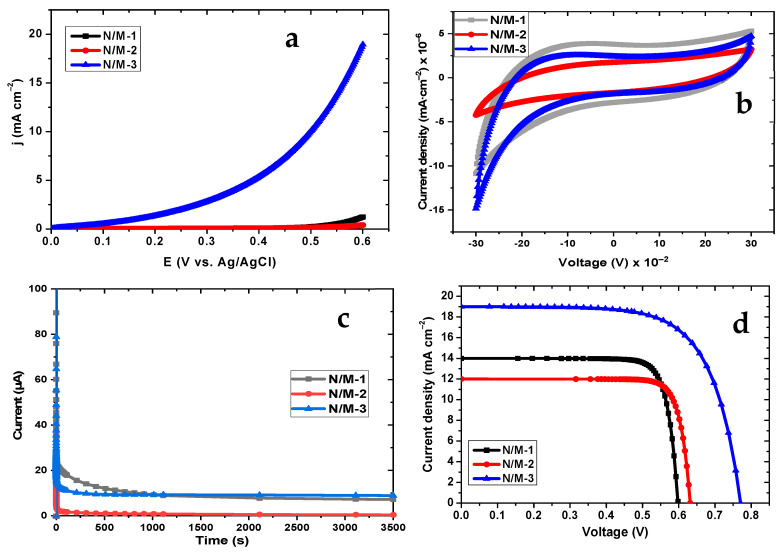
LSV (**a**), CV curve (**b**), CA (**c**), and I-V curve (**d**) of N/M-1, N/M-2, and N/M-3 cells.

To explore the reason why N/M-1, N/M-2, and N/M-3 particles work as active sites for power conversion, LSV was employed. In Figure 8a, the sensitiveness of the photocurrent response could be detected, showing the electron transfer process. The low current density and high onset potential of N/M-2 imply that the photocurrent response on the surface of N/M-2 is low [52]. The photocurrent response displays by the N/M-1 show a slight improvement in photocurrent response activity compared to the N/M-2. The hybridized N/M-3 cell device reveals the highest current density with a low potential onset, indicating that power conversion occurs more easily with N/M-3 because photons tend to be easily adsorbed on the surface of the MnS [52]. Likewise, the electrochemical characteristics of N/N-1, N/M-2, and N/M-3 cell devices were studied by varying the scan rate of 100 mV/s CV curves, as shown in Figure 8b. The cathodic and anodic peaks relating to the negative and positive current in the CV curves emanated from the reduction and oxidation activity of the H1-30 polysulfide electrolyte, which is mainly driven by charge recombination and Faradaic redox reactions in N/M-1, N/M-2, and N/M-3 devices. The disparity in charge recombination implies that both the outer and inner parts of the nanoporous material are involved in the photogeneration reaction [53]. The shortfall coming from the electron flows or connectivity means that there are interface faces caused by the oxides. This phenomenon has been reported for sulfide materials involving oxide composites, leading to a reduction in connectivity flow [53].

### 3.7. CA and I-V Analysis

To establish the electrochemical stability and durability of the N/M-1, N/M-2, and N/M-3 devices, a chronoamperometry (CA) test was conducted, as shown by the CA curve in Figure 8c using Equation (7).

(7) $$I=n\;F\;A\;c_{0}\sqrt{\frac{D}{\pi \;t}}$$

where *n* is the number of electrons transferred, *F* is Faraday’s Constant, *A* is the surface area of the working electrode, *C*_0_ is the initial concentration of the analyte, *D* is the diffusion coefficient of the analyte, and *t* is the time (s). In a few seconds from the initial period, there is a rapid current decrease for the N/M-1 and N/M-3 devices, indicating performance degradation, which could be caused by factors such as diffusion-controlled reactions, the irregular coating dissolution of cracks on the modified device, the loss of active support from the NiS passivative material, or the loss of an electrolyte, induced by evaporation [50]. Meanwhile, the N/M-2 modified device declines throughout the duration of the stability test, demonstrating a fully depleted device nature. The current reduction for the N/M-3 device slows down and remains at 8.88% of its initial current after 3500 s, showing better stability, while the N/M-1 device remains at 7.20% of the corresponding current. This finding implies that there is an interaction between the NiS loaded on the TiO_2_ and the MnS QD absorber. As a result of the anchoring effect of the protective layer, the stability of the modified N/N-3 improved significantly [52]. Figure 8d and Table 1 show the corresponding J-V curves and photovoltaic parameters of N/M-1, N/M-2, and N/M-3 modified cells using Equations (8) and (9).

(8) $$FF=V_{m}\times I_{m}/V_{OC}\times I_{SC}$$

(9) $$\eta =V_{OC}\times I_{SC}\times FF\times 100/P_{input}$$

where $V_{OC}$, $I_{SC}$, FF, and $P_{i}$ are the open-circuit voltage, short-circuit current, fill factor, and input power $(mW{cm}^{-2}$), respectively. As expected based on the trend from other results in this study, the N/M-3 displayed higher values, with a better performance of 9.94%, a fill factor (*FF*) of 0.68, a short-circuit current (*Jsc*) of 19 mA/cm^2^, and an open-circuit voltage (*Voc*) of 0.56 V. Apart from the slightly lower value for the *FF* of 0.59 in the N/M-1 device as compared to the N/M-2 with 0.63, the conversion efficiency is very close at 6.85% and 6.35%. This further shows that the NiS passivation layer promotes electron transport and enhances the performance of the modified cells, which is in agreement with our previous study with the MnS passivation layer [54].

## 4. Conclusions

The present study demonstrates that passivation of the NiS-MnS interface is a promising pathway for promoting a better conversion output in QSDCs. The size distributions of 2.92–6.39 nm with a polycrystalline nature correspond to (002) MnS and NiS. The change in *E_g_* values suggests that the passivation of NiS-MnS influences the energy levels of the cells by shifting the Fermi energy, leading to the narrowing of the band gap energy. The passivation of N/M-1 and N/M-3 devices has a slower or more resistive charge transfer process compared to that of N/M-2. The photochemical stability and durability test implies that the interaction between NiS and MnS improved the stability of N/N-3 compared to that of the other two devices. The N/M-3 with a short-circuit current of 19 mA cm^2^ and an enhanced power output *η* of 9.94% shows that the electron transport is enhanced by the multilayer cells. The findings from this study proselytize interfacial modification for future studies in QDSSCs.

## Figures and Tables

**Table 1:** I-V parameters for N/M-1, N/M-2, and N/M-3 devices; PS = present study.

Samples	*J_SC_* (mA/cm^2^)	*V_OC_* (V)	*FF*	*η* (%)	Ref.
N/M-1	14 ± 0.01	0.59 ± 0.0	0.83 ± 0.01	6.85 ± 0.01	PS
N/M-2	12 ± 0.01	0.63 ± 0.01	0.84 ± 0.02	6.35 ± 0.02	PS
N/M-3	19 ± 0.02	0.77 ± 0.0	0.68 ± 0.01	9.94 ± 0.01	PS
5% Mn-d-bS/CdS/CdSe/ZnS	16.11	0.52	0.47	4.25	[55]
TiO_2_/L-CdS/S-CdS	9.14	0.54	0.33	1.60	[56]
ZnO/CdS/CdSe/MnS(1 min)	13.74	0.60	0.44	3.70	[9]
CdSe with ZnS coating	12.2	0.53	0.31	2.02	[57]
ZnO/ZnS-MnS(0.100)/CdS	16.60	0.55	0.40	3.62	[33]
MnS/CdS/CdSe/ZnS	13.82	0.61	0.41	3.45	[58]
Mn:QD/Mn:ZnS	20.83	0.685	64.7	9.23	[59]
CdMnSe	19.15	0.58	0.57	6.33	[60]
CdS/Mn:CdSe	12.65	0.57	0.58	4.9	[61]
ZnO HMS/TiO_2_/TiCl_4_/10 min	14.57	0.45	0.46	2.99	[62]

Present study = PS.

## Data Availability

The original contributions presented in the study are included in the article, further inquiries can be directed to the corresponding authors.
